# Evaluation of Octenidine Dihydrochloride-Induced Cytotoxicity, Apoptosis, and Inflammatory Responses in Human Ocular Epithelial and Retinal Cells

**DOI:** 10.3390/biomedicines14010050

**Published:** 2025-12-25

**Authors:** Ihsan Hakki Ciftci, Asuman Deveci Ozkan, Gulay Erman, Imdat Kilbas, Ozlem Aydemir

**Affiliations:** 1 Faculty of Medicine, Department of Medical Microbiology, Sakarya University, Sakarya 54050, Turkey; 2 Department of Medical Biology, Faculty of Medicine, Sakarya University, Sakarya 54050, Turkey; 3 Health Services Education Research and Application Centre, Sakarya University, Sakarya 54050, Turkey; 4 Department of Medical Biochemistry, Institute of Health Science, Sakarya University, Sakarya 54050, Turkey; 5 Dental Prosthesis Technology Program, Vocational School of Health Services, Fenerbahce University, Istanbul 34758, Turkey; imdtklbs@gmail.com

**Keywords:** conjunctival diseases, eye diseases, cell culture techniques, bacterial eye infections

## Abstract

**Background/Objectives:** Octenidine dihydrochloride (OCT-D) is a broad-spectrum antiseptic with high chemical stability, low toxicity, and no reported microbial resistance, making it a strong candidate for use on mucosal surfaces. Despite increasing interest in its potential ophthalmic applications, limited data exist regarding its cellular effects on ocular tissues. This study aimed to investigate the cytotoxic, apoptotic, inflammatory, and transcriptional responses induced by OCT-D in human conjunctival (IOBA-NHC) and retinal pigment epithelial (ARPE-19) cells. **Methods:** Cells were exposed to varying concentrations of OCT-D, and viability was assessed using the WST-1 assay to determine IC_50_ and IC_50_/2 values. These concentrations were subsequently used in molecular assays. Pro-inflammatory cytokines (IL-6, IL-1β, TNF-α, IFN-γ) were quantified by ELISA. Apoptotic activation was evaluated through caspase-3/7 activity assays. Gene expression analysis of apoptotic (Bax, Bcl-2), DNA damage-related (ATM, Rad51), and inflammatory markers was performed using RT-qPCR. **Results:** OCT-D induced a marked, dose-dependent reduction in cell viability in both cell lines, with ARPE-19 showing greater sensitivity. Caspase-3/7 activity increased significantly at IC_50_ and IC_50_/2, confirming intrinsic apoptotic activation. OCT-D markedly suppressed the release of key inflammatory cytokines and downregulated transcription of inflammatory genes. RT-qPCR revealed upregulation of pro-apoptotic and DNA damage-associated genes, demonstrating coordinated activation of apoptotic and genomic stress pathways. **Conclusion:** OCT-D triggers integrated cytotoxic, apoptotic, and immunomodulatory responses in conjunctival and retinal epithelial cells. While these findings provide important mechanistic insights into OCT-D’s cellular effects, further studies using primary cells, advanced 3D ocular models, and disease-relevant systems are required to support its potential translational use in ophthalmology.

## 1. Introduction

Ocular microbial infections have become an increasing public health concern over the past two decades, leading to complications ranging from persistent inflammation to irreversible visual loss and blindness. Pathogenic microorganisms cause cellular and tissue damage through a variety of mechanisms, including toxin secretion by bacteria, cell lysis induced by viruses, and pro-inflammatory mediator release triggered by fungi. These events contribute to epithelial barrier disruption, tissue remodeling, and marked inflammatory responses that compromise ocular integrity [1,2,3]. Although conventional treatments—such as antibiotics, antifungals, antivirals, surgical interventions, and retinal prostheses—remain central to managing infectious ocular diseases, the rapid rise in antimicrobial resistance threatens the effectiveness of these therapeutic strategies. Current projections estimate that antimicrobial resistance may cause up to 300 million premature deaths and impose a global economic burden of nearly $100 trillion by 2050 [4]. Consequently, there is a growing need to identify alternative antimicrobial agents with improved safety profiles and reduced risk of resistance.

Octenidine dihydrochloride (OCT-D), an established mucosal antiseptic in several European countries, has attracted considerable attention due to its broad-spectrum antimicrobial efficacy, chemical stability, low toxicity, and lack of reported microbial resistance [5,6]. The cationic nature of OCT-D enables strong binding to negatively charged surfaces such as microbial cells and human epithelial membranes [7]. This property facilitates a long-lasting biocidal effect by preventing microbial regrowth after disinfection. In addition, OCT-D has been shown to retain antiseptic activity even against microorganisms protected within biofilms [8]. The primary reasons why bacteria display very low or no resistance to OCT include its multi-target mode of action, its ability to disrupt essential cellular structures, and its rapid bactericidal activity, which together reduce the likelihood of resistance development compared to conventional antibiotics. This favorable resistance profile has increased interest in OCT-D as a potential ophthalmic antiseptic. Given its membrane-active mechanism of action, OCT-D is increasingly evaluated for potential ophthalmic applications. However, a detailed understanding of its cellular effects on ocular tissues is essential to ensure its safety before clinical use.

Conjunctival epithelial cells constitute the first-line barrier on the ocular surface and play a key role in maintaining epithelial integrity and coordinating immune responses [9]. In vitro studies using human ocular epithelial models are therefore indispensable for assessing the safety and cellular effects of candidate antiseptics. In this context, IOBA-NHC (conjunctival epithelial cells) represents a widely used model for ocular surface research, whereas ARPE-19, although not an ocular surface cell line, serves as a well-established model for retinal pigment epithelial function and provides additional insight into potential off-target or deeper-tissue cellular responses relevant to ophthalmic safety testing [10].

This study comprehensively investigated the cellular effects of OCT-D by evaluating its cytotoxic, apoptotic, inflammatory, and transcriptional impacts on IOBA-NHC and ARPE-19 cells. Specifically, we aimed to (i) determine the dose-dependent effects of OCT-D on cell viability, (ii) quantify inflammatory cytokine responses using ELISA and RT-qPCR, and (iii) assess its influence on apoptotic pathways through caspase-3/7 activation and gene expression profiling. These analyses provide a multi-level understanding of how OCT-D modulates ocular epithelial and retinal epithelial cell responses.

## 2. Materials and Methods

### 2.1. Cell Culture Conditions

The study was carried out using two human ocular cell lines: IOBA-NHC (normal human conjunctival epithelial cells) and ARPE-19 (adult retinal pigment epithelial cells). Both cell lines were maintained according to the supplier’s guidelines in Dulbecco’s Modified Eagle Medium (DMEM) medium supplemented with 10% fetal bovine serum (FBS) and 1% penicillin–streptomycin. Cultures were incubated at 37 °C in a humidified atmosphere containing 5% CO_2_ [11].

### 2.2. Cell Viability Assay

IOBA-NHC and ARPE-19 cells were seeded into 96-well plates at a density of 2 × 10^4^ cells/mL and allowed to adhere overnight. The cells were subsequently exposed to various concentrations of OCT-D (0.4–0.00625%) for 12–24 h. Following treatment, 10 µL of WST-1 reagent was added to each well and the plates were incubated at 37 °C for an additional 1–4 h. Optical density was then measured using a microplate reader at 460–620 nm. Cell viability was calculated relative to the untreated negative control, which was considered as 100% viability. Based on these analyses, the determined IC_50_ and IC_50_/2 concentrations were selected and applied in all subsequent experimental assays.

### 2.3. Cytokine Quantification by Enzyme-Llinked Immunosorbent Assay (ELISA)

To evaluate the inflammatory response induced by OCT-D, protein levels of IL-1β (E0143Hu), IFN-γ (E0105Hu), TNF-α (E0082Hu), and IL-6 (E0090Hu) were quantified in IOBA-NHC and ARPE-19 cells using commercially available ELISA kits (BT Lab, Shanghai, China). Cell culture supernatants were collected following treatment and processed according to the manufacturers’ instructions. Briefly, standards and samples were added to pre-coated plates and incubated, followed by sequential incubation with biotinylated detection antibodies and streptavidin–HRP conjugate. After washing steps, substrate solutions were added, and the reaction was terminated with stop solution. Absorbance was measured at 450 nm using a microplate reader, and cytokine concentrations were calculated from standard curves generated for each analyte. Quantification was performed by converting optical density values to concentration units based on the corresponding standard curve equations and R^2^ values [12].

### 2.4. Caspase Activity Measurement

Caspase activity was evaluated using cell lysates prepared from treated IO-BA-NHC and ARPE-19 cells. Following the experimental treatment procedures, cells (1 × 10^6^ cells/mL) were washed twice with ice-cold PBS to remove residual medium and resuspended in 50 µL of cold lysis buffer. The suspension was briefly vortexed and incubated on ice for 30 min to ensure complete and uniform cell lysis. During this period, samples were gently mixed at regular intervals. Lysates were then clarified by centrifugation at 12,000× *g* for 10 min at 4 °C, and the resulting supernatants, containing soluble cytosolic proteins, were carefully collected and kept on ice until analysis. For the enzymatic reaction, 20–50 µL of each clarified lysate was transferred to a 96-well plate and combined with reaction buffer and the caspase-specific chromogenic substrate (final concentration: 100 µM), resulting in a total reaction volume of 200 µL per well. Plates were incubated at 37 °C for 1–2 h under protected conditions. Caspase activity was quantified by measuring the release of p-nitroaniline (pNA), the chromogenic cleavage product, at 405 nm using a microplate reader [13,14].

### 2.5. Gene Expression Analysis by Real-Time Polymerase Chain Reaction (RT-PCR) Analysis

To investigate the apoptotic, DNA damage-related, and inflammatory effects of OCT-D at the molecular level, mRNA expression levels of *BAX*, *BCL-2*, *ATM*, *RAD51*, *IL-1β*, *IFN-γ*, *TNF-α*, and *IL-6* were quantified using real-time PCR (RT-PCR) method. Total RNA was extracted from IOBA-NHC and ARPE-19 cells using a commercially available RNA isolation kit, and RNA concentration and purity were assessed by spectrophotometry (NanoDrop, Waltham, MA, USA). High-quality RNA samples were subsequently reverse-transcribed to cDNA using a standard cDNA synthesis kit following the manufacturer’s instructions. Quantitative PCR was performed using gene-specific primers and a SYBR Green detection system on a real-time PCR instrument (Bio-Rad Laboratories, Hercules, CA, USA). All reactions were carried out in appropriate cycling conditions recommended for SYBR Green chemistry. Relative gene expression levels were calculated using the ΔΔCt method, with internal housekeeping gene (β-actin) used for normalization [15,16]. In this study, primer sets were designed using NCBI Primer-Blast software The primers used are as Table 1.

### 2.6. Statistical Analysis

Statistical analyses were performed using GraphPad Prism (V.10.6.1; GraphPad Software, San Diego, CA, USA). Data were expressed as mean ± standard deviation (SD). Differences between groups were evaluated using one-way ANOVA, followed by appropriate post hoc tests when applicable. IC_50_ values were calculated from the cell viability curves using a nonlinear regression model (variable slope, four-parameter logistic curve; sigmoidal dose–response) in GraphPad Prism. A *p*-value < 0.05 was considered statistically significant [17,18,19].

## 3. Results

### 3.1. OCT-D Induces Dose-Dependent Reductions in Viability of IOBA-NHC and ARPE-19 Cells

**Figure 1:**
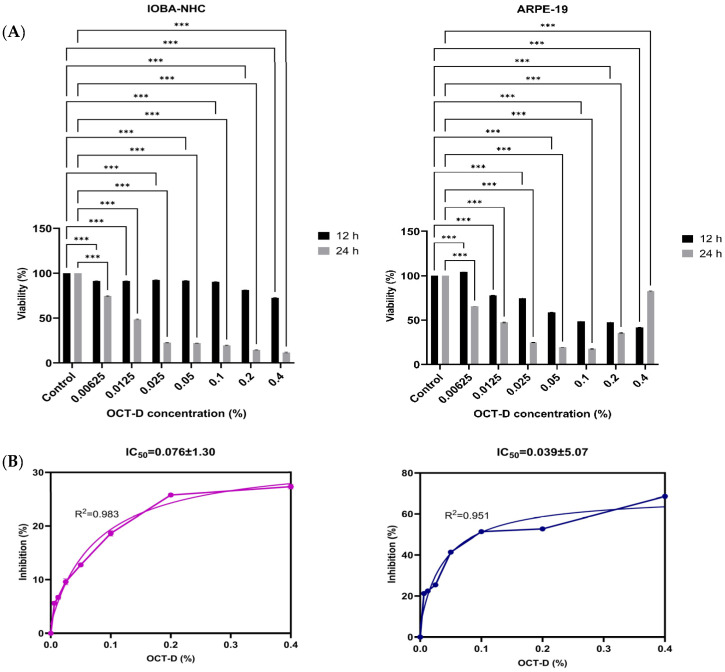
Effects of OCT-D on cell viability and IC_50_ determination in IOBA-NHC and ARPE-19 cells. (**A**) Cell viability (%) of IOBA-NHC and ARPE-19 cells following treatment with increasing concentrations of OCT-D for 12 and 24 h. (**B**) Dose–response inhibition curve and IC_50_ determination for OCT-D in IOBA-NHC and ARPE-19 cells. Data are presented as mean ± SD from triplicate experiments (*** *p* < 0.001).

WST-1 viability analysis revealed that OCT-D [0.4%, 0.2%, 0.1% (commercial concentration), 0.05%, 0.025%, 0.0125%, and 0.00625%] exerted a marked, dose-dependent cytotoxic effect on both human ocular cell lines (Figure 1). In IOBA-NHC cells, viability significantly decreased at all tested concentrations compared with the control after both 12 h and 24 h of exposure (*p* < 0.001). A similar pattern was observed in ARPE-19 cells, in which OCT-D treatment resulted in substantial reductions in cell viability across the entire concentration range (*p* < 0.001). The decrease was more pronounced at 24 h, indicating a time-dependent effect (Figure 1A). Dose–response inhibition curves confirmed the cytotoxic potential of OCT-D, yielding IC_50_ values of 0.076 ± 1.30% for IOBA-NHC and 0.039 ± 5.07% for ARPE-19 cells (Figure 1B). These findings indicate that ARPE-19 cells were more sensitive to OCT-D exposure compared with IOBA-NHC cells. Based on the viability results, the IC_50_ concentrations determined at 12 h, as well as their half concentrations (IC_50/2_), were selected and applied in all subsequent experimental assays.

### 3.2. OCT-D Suppresses Pro-Inflammatory Cytokine Production in IOBA-NHC and ARPE-19 Cells

**Figure 2:**
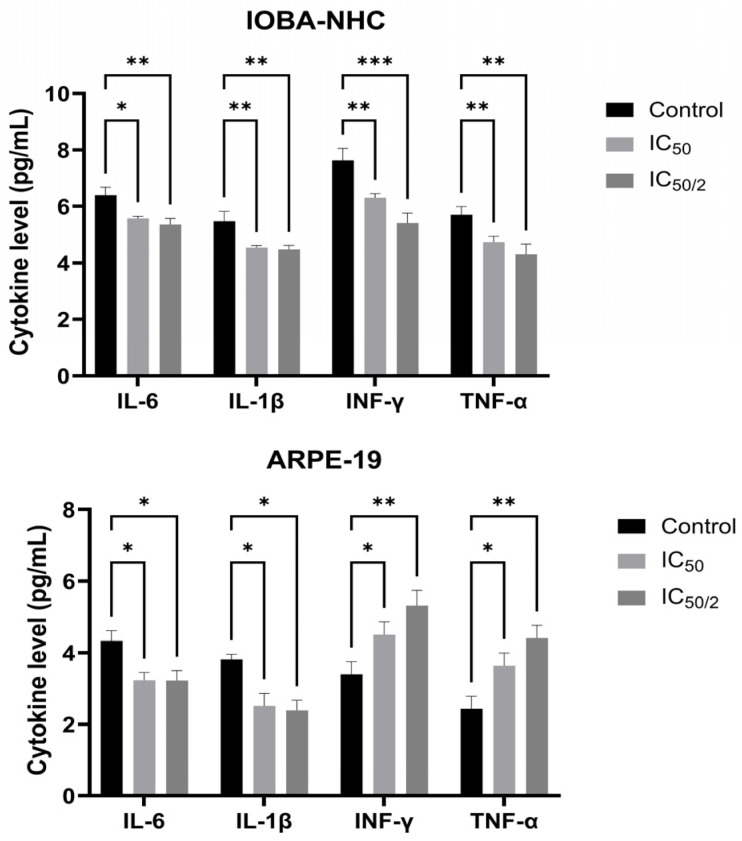
Effects of OCT-D on pro-inflammatory cytokine levels in IOBA-NHC and ARPE-19 cells. Protein levels of IL-6, IL-1β, IFN-γ, and TNF-α were quantified by ELISA in IOBA-NHC and ARPE-19 cells. Data are presented as mean ± SD from triplicate experiments (* *p* < 0.05, ** *p* < 0.01, *** *p* < 0.001).

ELISA analysis demonstrated that OCT-D significantly suppressed the production of key pro-inflammatory cytokines in both ocular cell lines (Figure 2). In IOBA-NHC cells, treatment with IC_50_ and IC_50_/2 concentrations of OCT-D resulted in a marked reduction in IL-6, IL-1β, IFN-γ, and TNF-α levels compared with the control group (*p* < 0.05 to *p* < 0.001, Figure 2). ARPE-19 cells showed a similar response, with all cytokines exhibiting a significant, concentration-dependent decrease following OCT-D exposure. Notably, ARPE-19 cells appeared slightly more sensitive, displaying a more pronounced cytokine reduction at IC_50_ treatment (Figure 2). These results indicate that OCT-D exerts a clear anti-inflammatory effect in ocular epithelial and retinal pigment epithelial cells.

### 3.3. OCT-D Induces Caspase-Dependent Apoptotic Activation in IOBA-NHC and ARPE-19 Cells

**Figure 3:**
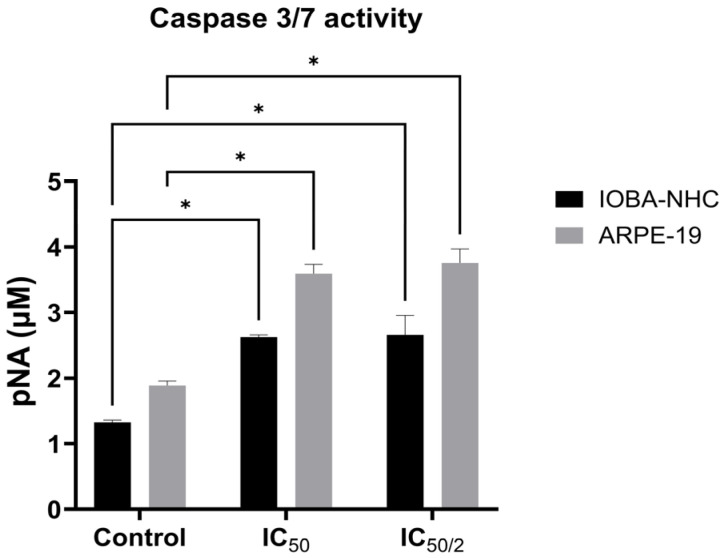
Caspase-3/7 activity in IOBA-NHC and ARPE-19 cells following OCT-D treatment. Data are presented as mean ± SD (* *p* < 0.05).

Caspase-3/7 analysis revealed that OCT-D markedly enhanced apoptotic signaling in both ocular cell lines (Figure 3). Compared with the control, IOBA-NHC and ARPE-19 cells exhibited significantly increased caspase activity at both IC_50_ and IC_50_/2 concentrations (*p* < 0.05, Figure 3). ARPE-19 cells showed a stronger apoptotic response, demonstrating higher pNA release than IOBA-NHC cells at both treatment levels (Figure 3). These findings indicate that OCT-D effectively triggers caspase-dependent apoptosis, with retinal epithelial cells demonstrating greater sensitivity to OCT-D-induced apoptotic activation.

### 3.4. OCT-D Modulates Apoptotic, DNA Damage-Related, and Inflammatory Gene Expression in IOBA-NHC and ARPE-19 Cells

**Figure 4:**
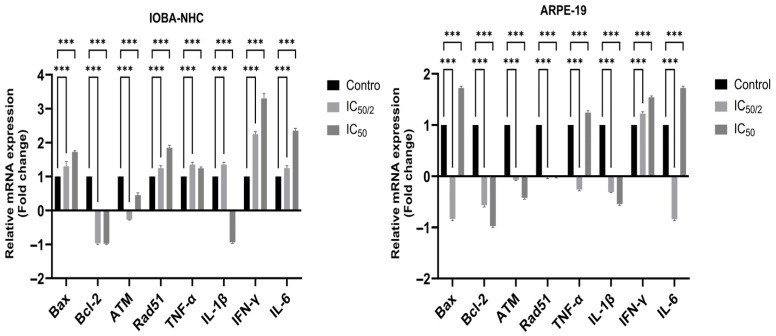
Effects of OCT-D on apoptotic, DNA damage-related, and inflammatory gene expression in IOBA-NHC and ARPE-19 cells. Data are presented as fold change (mean ± SD, *** *p* < 0.001).

RT-PCR analysis revealed that OCT-D induced significant alterations in the expression of genes associated with apoptosis, DNA damage response, and inflammation in both ocular cell lines (Figure 4). In IOBA-NHC cells, OCT-D treatment markedly upregulated the pro-apoptotic gene *Bax* while downregulating the anti-apoptotic gene *Bcl-2*, indicating a shift toward apoptotic activation. DNA damage-related regulators *ATM* and *Rad51* were also significantly increased, supporting OCT-D-induced genomic stress. Additionally, OCT-D reduced the expression of inflammatory cytokine genes (*TNF-α*, *IL-1β*, *IFN-γ*, *IL-6*), consistent with its anti-inflammatory effect observed at the protein level. A similar expression profile was detected in ARPE-19 cells, where IC_50_ and IC_50_/2 treatments led to pronounced downregulation of *Bcl-2* and inflammatory markers, accompanied by upregulation of *Bax* and DNA damage-associated genes (Figure 4). Compared with IOBA-NHC cells, ARPE-19 cells exhibited a more prominent reduction in inflammatory gene expression, suggesting greater sensitivity to OCT-D. Overall, these findings indicate that OCT-D promotes apoptotic signaling, induces DNA damage response pathways, and suppresses inflammatory gene expression at the transcriptional level.

## 4. Discussion

Studies on the cytotoxic effects of OCT-D have indicated lower toxicity on fibroblasts and epithelial cells than other commonly used antiseptics, such as chlorhexidine. In this context, it can be said that OCT-D has less adverse effects on cell metabolism and viability than other antiseptics and could potentially be a safer alternative [20,21,22]. As a result of the findings obtained from WST-1 analysis, it was observed that there were significant concentration and time-dependent changes in OCT-D in IOBA-NHC and ARPE-19 cell lines. Statistically significant differences were observed between the viability rates at 12 and 24 h at all concentrations except 0.00625% OCT-D.

Increased caspase 3/7 activity is an important indicator of the apoptotic process. Stahl et al. observed concentration-dependent apoptotic responses in their study examining the effects of various antiseptic agents on epithelial cells [23]. Similarly, our study observed a dose-dependent effect, particularly in ARPE-19 cells, with significant apoptotic activation at the IC_50_ concentration. This effect was more limited in IOBA-NHC cells. This variable effect of OCT-D on cell types in different eye regions highlights the importance of a tissue-specific approach in antiseptic selection. Müller and Kramer, in their study comparing the cytotoxic effects of antiseptic agents, emphasized the importance of establishing concentration-dependent safety profiles [24]. Yanık et al. (2011) noted that in vitro studies evaluating the effects of antiseptics on tissue may not fully translate to clinical practice but can provide important preliminary data [25]. In future studies, elucidating the molecular mechanisms of this apoptotic effect and validating it in in vivo models will contribute to a better understanding of the safety profile of OCT-D for ophthalmic use.

Our study observed that the effect of OCT-D on cytokine release exhibits a dose-dependent pattern. It showed significant changes in ARPE-19 cells, particularly at the IC_50_ concentration. Koizumi et al. have shown that antiseptic agents can trigger different inflammatory responses in ocular cells depending on the dose, and that the release of pro-inflammatory cytokines can be increased, especially at higher concentrations [26]. Similarly, their study examining ocular antiseptics dose-dependent cytotoxic and inflammatory effects reported that concentrations around the IC_50_ can trigger significant inflammatory responses [27]. Baudouin et al. reported that preservatives in eye drops may dose-dependently increase the expression of pro-inflammatory cytokines in ocular surface cells, contributing to ocular irritation and inflammation [28]. Similarly, in our study, changes in cytokine release were observed with OCT-D, particularly at the IC_50_ dose, consistent with literature data. They reported that ARPE-19 cells increased inflammatory cytokine production when exposed to various irritants, and IL-1β release was particularly significantly elevated [29]. Similarly, Moschos et al. (2012) demonstrated that some antimicrobial agents induced the expression of IL-1β and other pro-inflammatory cytokines in ARPE-19 cell [30]. Yang et al. reported that an increase in IL-1β in RPE cells is associated with NLRP3 inflammasome activation, which may contribute to retinal inflammation [31]. RT-PCR findings were also consistent with the ELISA results presented above. The increased IL-1β release observed in ARPE-19 cells by OCT-D in our study is consistent with literature findings and suggests that this antiseptic could potentially cause inflammasome activation.

Paulsen et al. reported that conjunctival epithelial cells exhibit cell-type-specific inflammatory responses when exposed to antiseptic agents, and that these responses may differ from other ocular cell lines such as ARPE-19 [32]. De Saint Jean et al. reported that a dose-dependent pattern can be observed in the inflammatory response of conjunctival epithelial cells, and that while cytokine release tends to increase at specific concentrations, it may not reach statistical significance [33]. Paulsen et al. reported that ocular surface antiseptics do not cause a significant increase in IL-6 production at low concentrations, but may tend to increase at higher concentrations [34]. Similarly, they noted that some antimicrobial agents minimally affect IL-6 release in ARPE-19 cells at low concentrations but may cause an increase at doses of IC_50_ and above [27]. Debbasch et al. (2001), in their study examining the effects of preservatives on IL-6 release in conjunctival epithelial cells, reported that the cytokine response was dose-dependent but did not always reach statistical significance [35]. No significant increase in IL-1β and IL-6 levels was observed after OCT-D application in IOBA-NHC cells. This finding suggests that conjunctival epithelial cells exhibit a different inflammatory response profile than retinal pigment epithelial cells. RT-PCR findings revealed a statistically significant increase in IL-6 release in ARPE-19 cells in the 1/2 IC_50_ and IC_50_ groups. In our study, we observed that the effect of OCT-D on IL-6 expression differed between ARPE-19 and IOBA-NHC cells. The dose-dependent increase was more pronounced in ARPE-19 cells, while this increase was more limited in IOBA-NHC cells. This finding is consistent with studies comparing the cytokine responses of different ocular cell types [36]. The researchers reported that ARPE-19 cells respond more strongly to inflammatory stimuli than conjunctival epithelial cells. Similarly, they noted that IL-6 production in IOBA-NHC cells is more controlled than other inflammatory cytokines and shows minimal changes in response to certain irritants [37]. In their original study characterizing IOBA-NHC cells, they emphasized that the IL-6 expression profile of these cells may differ from other ocular cell lines and exhibit a more stable expression pattern in response to various stimuli [38]. Our study’s limited IL-6 response in IOBA-NHC cells is consistent with the literature. However, RT-PCR found a statistically significant increase in IL-6 release in IOBA-NHC cells in the 1/2 IC_50_ and IC_50_ groups. The dose-dependent IL-6 increase we observed in both ARPE-19 and IOBA-NHC cells is consistent with various studies in the literature [39].

TNF-α is a key cytokine in intracellular signaling and initiation of inflammation and plays an important role in retinal degeneration and inflammatory eye diseases [40,41]. A statistically significant increase in TNF-α levels was observed after OCT-D application in ARPE-19 cells. Similarly, a dose-dependent increase in TNF-α levels was observed in IOBA-NHC cells and was significant at the IC_50_ dose. However, basal TNF-α levels in this cell line were higher in the control group than in ARPE-19 cells, suggesting that conjunctival epithelial cells are more susceptible to innate immune mechanisms [42]. The TNF-α findings obtained by RT-PCR are consistent with the ELISA results. The findings indicate that OCT-D may initiate the inflammatory process by increasing TNF-α production in both retinal and conjunctival-derived cells; Therefore, immune side effects, as well as cytotoxic effects, should be taken into consideration.

This study examined changes in IL-4 levels to understand the immunomodulatory effects of OCT-D application on epithelial cells. Significant and statistically significant increases in IL-4 levels were observed after OCT-D in ARPE-19 cells, indicating that T-cell-derived cytokine production was triggered. IL-4 is a cytokine released from Th2 cells and plays a role in humoral immunity and anti-inflammatory processes [43]. Although the increase in IL-4 in IOBA-NHC cells was not statistically significant, it is biologically significant. High basal IL-4 levels in this cell line suggest that the conjunctival epithelium has a tolerogenic or immune-active microenvironment [44]. RT-PCR findings are consistent with ELISA results. An increase in IL-4 was observed in both cell types, with the increase being more pronounced in ARPE-19 cells, both statistically and biologically. This suggests that retinal cells may develop complex Th2-type immune responses to OCT-D.

Our study demonstrated that OCT-D administration affects IL-10 expression in ocular epithelial cell lines differently. A significant decrease in IL-10 levels was observed with increasing dose in ARPE-19 cells, indicating a weakening of anti-inflammatory mechanisms. In contrast, IL-10 levels were significantly increased in IOBA-NHC cells, suggesting that these cells have developed an active immune balancing strategy against pharmacological stress. The primary role of IL-10 in the immune system is the inhibition of pro-inflammatory cytokines and regulating immune cells [45]. RT-PCR findings are consistent with ELISA results. Similarly, other in vitro studies have reported that conjunctival cells increase IL-10 production in response to oxidative and inflammatory stress [46,47], and this increase contributes to the preservation of ocular surface integrity by limiting excessive inflammation on mucosal surfaces [48]. The decrease in IL-10 levels in ARPE-19 cells suggests that OCT-D disrupts the immune balance and creates a pro-inflammatory tendency; in contrast, the increased IL-10 in IOBA-NHC cells suggests that a different regulatory mechanism operates that activates cytoprotective effects [49]. The inflammatory suppressive effects of IL-10 are mediated through intracellular signaling pathways and cytokine levels [50]. In this context, the increase in IL-10 observed in IOBA-NHC cells indirectly suggests activation of the NF-κB or STAT3 pathways and demonstrates that ocular epithelia possess cell-type-specific properties in immune regulation [51].

One limitation of this study is the use of IOBA-NHC cells, which are spontaneously immortalized conjunctival epithelial cells rather than primary cultures. Although widely used and well-characterized in ocular surface research, immortalized cell lines may exhibit altered sensitivity to cytotoxic stimuli compared with primary conjunctival cells. Therefore, the cellular responses observed here may not fully reflect native conjunctival physiology. Future studies employing primary conjunctival epithelial cells or advanced 3D ocular surface models will be necessary to validate and extend the present findings. On the other hand, another limitation of the present study is the absence of classical apoptosis–necrosis discrimination assays such as MTT/MTS or Annexin V/PI flow cytometry. Although caspase-3/7 activation, Bax/Bcl-2 gene regulation, DNA damage markers, and viability data collectively indicate a predominantly apoptotic response to OCT-D, these methods do not fully distinguish apoptotic from necrotic cell death. Future studies incorporating flow cytometric Annexin V/PI profiling, necrosis-specific markers, and morphological analyses will be required to more comprehensively characterize the mode of cell death induced by OCT-D.

## 5. Conclusions

In conclusion, this study comprehensively evaluated the cytotoxic, apoptotic, inflammatory, and immunomodulatory effects of OCT-D in IOBA-NHC and ARPE-19 ocular epithelial cell lines. When the results are considered together, a clear mechanistic sequence emerges: the dose-dependent reduction in cell viability indicates the onset of cellular stress, which is followed by activation of caspase-3/7, demonstrating progression toward intrinsic apoptosis. Parallel suppression of key pro-inflammatory cytokines (*IL-6*, *IL-1β*, *TNF-α*, *and IFN-γ*), together with transcriptional downregulation of inflammatory genes and modulation of apoptotic and DNA damage-related markers (*Bax*, *Bcl-2*, *ATM*, *Rad51*), reveals that OCT-D also exerts significant immunomodulatory and transcriptional effects in a cell type-dependent manner. These coordinated alterations indicate that OCT-D triggers an integrated cellular response involving cytotoxicity, apoptosis, DNA damage signaling, and inflammatory regulation in ocular epithelial cells.

Although these findings provide important mechanistic insights, further studies are needed to support the translational relevance of OCT-D. Future work should incorporate broader dose–response ranges, time-series analyses, and evaluations of additional molecular regulators such as NF-κB, STAT3, and TGF-β pathways. More physiologically relevant models—including conjunctival–retinal co-cultures, 3D ocular surface systems, and disease models such as dry eye, retinal inflammation, or conjunctivitis—will be essential to better characterize immune homeostasis and validate these in vitro observations.

## Figures and Tables

**Table 1:** RT-qPCR primer sequences used in this study.

Gene	Forward Primer (5′ → 3′)	Reverse Primer (5′ → 3′)
GAPDH	CCTGCGACTTCAACAGCAAC	CTGCTTCACCTCCCCATACA
IL-1β	GCTGGAGGTGAGTCCATCAG	GTGATAACGGTGGCCTGACA
IL-6	CCCCAATTTCCAATGCTCTCC	CGCACTAGGTTTGCCGAGTA
TNF-α	ATCCATCTTTGCGGAGGC	GGGGGAGAGGTAGGGATGTT
IL-10	TTGAACCACCCGGCATCTAC	CCAAGGAGTTGCTCCCGTTA
IL-4	CCATATCCACGGATGCGACA	AAGCCCGAAAGAGTCTCTGC

## Data Availability

The data presented in this study are available in the article. Further inquiries can be directed to the corresponding author.
